# Strong association between vertebral endplate defect and Modic change in the general population

**DOI:** 10.1038/s41598-018-34933-3

**Published:** 2018-11-09

**Authors:** Juhani H. Määttä, Marinko Rade, Maxim B. Freidin, Olavi Airaksinen, Jaro Karppinen, Frances M. K. Williams

**Affiliations:** 1 Medical Research Center Oulu, Oulu University Hospital and University of Oulu, P.O. Box 8000, 90014 Oulu, Finland; 2 Orton Rehabilitation Centre, Tenholantie 10, 00280 Helsinki, Finland; 3 Department of Twin Research and Genetic Epidemiology, King’s College London, London, SE1 7EH UK; 4 Department of Physical and Rehabilitation Medicine, Kuopio University Hospital, P.O. Box 1607, 70211 Kuopio, Finland; 5 Josip Juraj Strossmayer University of Osijek, Faculty of Medicine, Orthopaedic and Rehabilitation Hospital “Prim. dr. Martin Horvat”, Luigi Monti street n.2, 52210 Rovinj, Croatia; 6 Juraj Dobrila University of Pula, Department of Natural and Health Studies, Zagrebacka 30, 52100 Pula, Croatia; 7 Finnish Institute of Occupational Health, Aapistie 1, 90220 Oulu, Finland

## Abstract

Modic change (MC) is considered an independent risk factor for low back pain (LBP) but its aetiology remains unclear. In this cross-sectional, large-scale population-based study we sought to characterise associations between endplate defect (ED) and MC in a population sample of broad age range. The study population consisted of 831 twin volunteers (including 4155 discs and 8310 endplates) from TwinsUK. Lumbar T2-weighted MR images were coded for ED and MC. Total endplate (TEP) score was calculated at each intervertebral disc while receiver operating curves (ROC) were calculated to define critical endplate values predictive of MC. MC was detected in 32.1% of the subjects, with a significantly higher prevalence at lower lumbar levels (3.5% at L1/2-L3/4 vs. 15.9% at L4/5-L5/S1, p < 0.001). TEP score was strongly and independently associated with MC at each lumbar level (risk estimates from 1.49 to 2.44; all p ≤ 0.001) after adjustment for age, sex, BMI and twin pairing. ROC analysis showed a TEP score cut-off of 6 above which there was a significantly higher prevalence of MC. In conclusion, ED were strongly associated with MC at every lumbar level. These findings support the hypothesis that endplate defect is a major initiating factor for the cascade of events that may include disc degeneration (DD) and MC.

## Introduction

Globally, low back pain (LBP) is among the leading causes of disability^1^ and is considered the largest single cause of disability in Western society, with lifetime prevalence of non-specific LBP estimated at 60–70% in industrialized countries^2^. With increasing patient demand in secondary and tertiary care, requesting appointments with rheumatologists, physical and rehabilitation medicine specialists and surgeons, LBP represents a highly relevant public health issue with enormous healthcare and socioeconomic costs^3^. The precise causes of such a burden have still not been clearly defined.

Modic change (MC) describes MR image signal change in subchondral and vertebral bone marrow adjacent to a vertebral body endplate. As visualized on T1- and T2-weighted MR imaging, MC can be divided into three different subtypes: Type 1, 2 and 3^4–6^. MC is rarely observed adjacent to a healthy disc^7^ and tends to be associated with structural change such as disc degeneration (DD)^7–9^, disc herniation^7,8,10^, Schmorl’s nodes^7^ and vertebral endplate defect^11^. Similar to association of intervertebral DD with LBP^12,13^, also MC is associated with it^14,15^, and is now considered to be an independent risk factor for LBP^16^.

Different theories have been suggested regarding the aetiology of these conditions, but the pathophysiology of MC is still not fully understood, and the role of structural bony defects as an initiating factor for MC is not confirmed. Lately, clinical studies have focused attention on endplate defects as having a possible role in the aetiopathogenesis of MC^17,18^.

Rajasekaran *et al*.^19^ have reported that the presence of endplate defect was associated with DD in a clinical sample of 47 patients and 26 volunteers. Endplate defect evaluated on T1-weighted MR scans was classified into six categories according to severity of damage (type 1 to type 6), and total endplate (TEP) score was derived. Strong correlation between progressive grades of endplate defect and disc degeneration was shown, but unfortunately MC was not considered independently in the statistical analysis.

In a previous study from TwinsUK we have shown a strong association between endplate defect and DD in a large population based sample of volunteer twins^20^. As the association between endplate defect and DD was strong across the age spectrum, and as the endplate is positioned exactly between the intervertebral disc and the vertebral body, we sought to explore whether an additional association exists between endplate defect and MC.

## Methods

### Study population

The study population was part of the TwinsUK register of King’s College London (www.twinsuk.ac.uk)^12^. Clinical information on sex, body mass index (BMI), and lumbar DD had been collected previously. All subjects have signed an informed consent form. The study was approved by St. Thomas’ Hospital Ethics Committee and is in accordance with Declaration of Helsinki.

### Magnetic resonance imaging and grading

Details of the MR scans, and grading of MRI findings, have been described previously^21^. Briefly, T2-weighted MR scans were performed on volunteer subjects unselected for LBP, using a Siemens (Munich, Germany) 1.0-T superconducting magnet. Sagittal T2-weighted images were obtained using a fast spin-echo sequence of time to recovery (TR)/time to echo (TE) 5000–4500/112 msec, with a slice thickness of 4 mm. Grading was performed on T2-weighted images.

#### Grading of endplate defect

**Figure 1:**
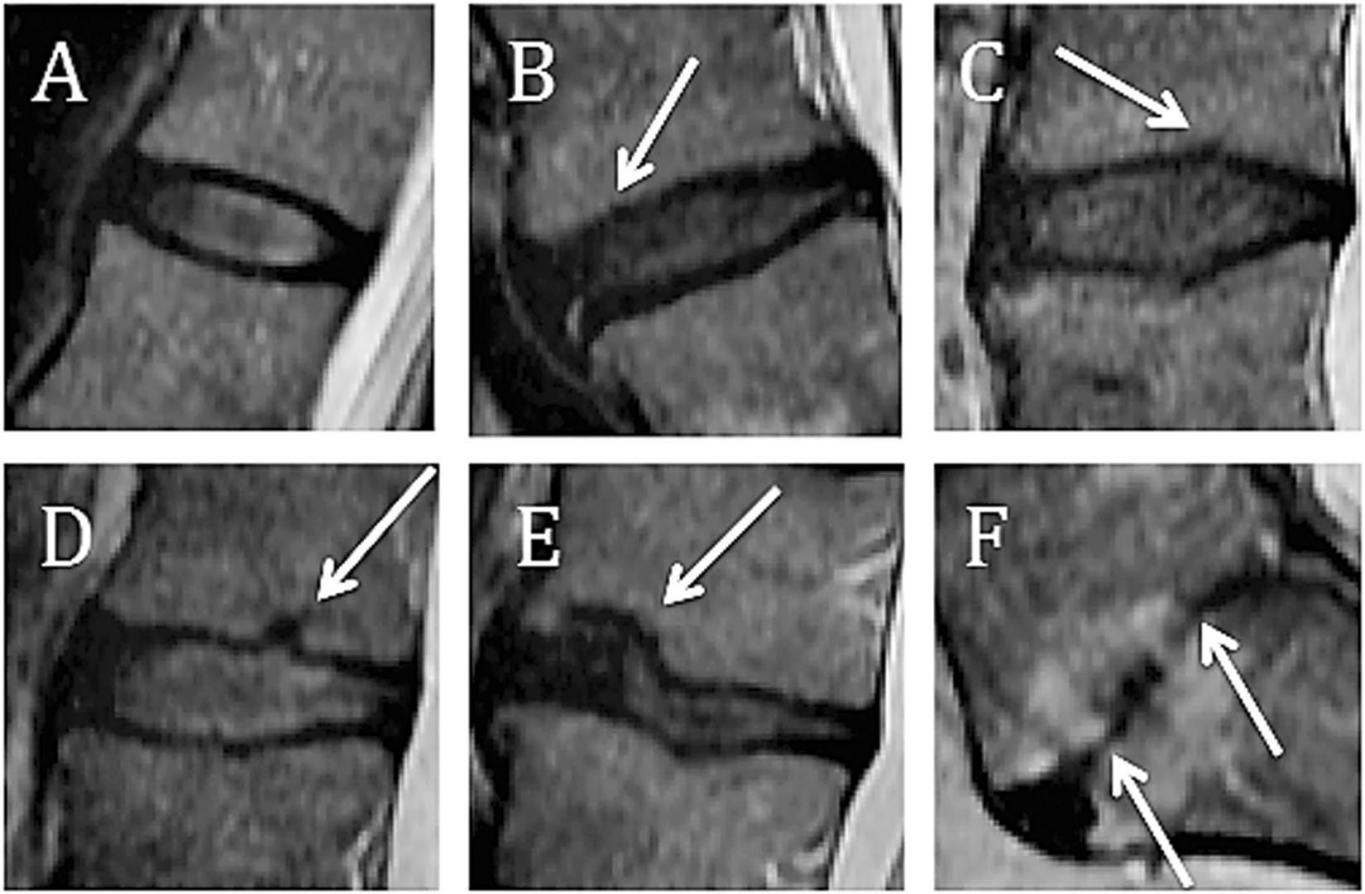
Endplate grading. (**A**) Grade 1: Normal endplate, no breaks or defects, (**B**) Grade 2: Focal thinning (white arrow) of the endplate, no breaks or defects, (**C**) Grade 3: Focal disc marrow contacts (white arrow), but with maintained endplate contour, (**D**) Grade 4: Endplate defects up to 25% of the endplate area (white arrow), (**E**) Grade 5: Endplate defects up to 50% of the endplate area (white arrow), (**F**) Grade 6: Extensive damaged endplates up to total destruction (white arrows). From Rade M. *et al*. Vertebral endplate defect as initiating factor in intervertebral disc degeneration: Strong association between endplate defect and disc degeneration in the general population. *Spine* **43**, 412–419 (2018). With permission.

As reported previously^20^, authors JHM and MR coded endplate defects in the baseline MRI scans from all 831 subjects (including 4155 discs and 8310 endplates). Endplate defect was evaluated on a scale of 1–6 (Table 1, Fig. 1) according to Rajasekaran *et al*.^19^ and total endplate scores for each disc were constructed by summing the endplate defect score of both rostral and caudal endplates in each functional spine unit^19^. Inter-rater agreement for the coding of endplate defect was calculated using Cohen’s weighted kappa and Pearson’s correlation.

**Table 1:** Endplate defect score.

Grade 1	Normal endplate, no breaks or defects
Grade 2	Focal thinning of the endplate, no breaks or defects
Grade 3	Focal disc marrow contacts, but with maintained endplate contour
Grade 4	Endplate defects up to 25% of the endplate area
Grade 5	Endplate defects up to 50% of the endplate area
Grade 6	Extensive damaged endplates up to total destruction

Agreement phase: An initial training phase was held in which an inter-rater agreement on endplate defect detection and grading of ≥0.85 was reached on at least 100 subjects and 1000 endplates, as reported previously^20^. Disagreements on coding scores were settled by discussion and consensus.

#### Grading of Modic change

The coding of MC was performed previously and has been described in detail elsewhere^22^. Briefly, a single observer (JHM) assessed all images without any prior knowledge of the clinical status. Inter-rater reliability was calculated when the second reader (SW) assessed randomly selected subset of images (n = 50). For the purpose of this study, MC was coded as absent (grade 0) or present (grade 1) for vertebral bone marrow adjacent to each endplate separately. MC detected only in one sagittal slice was not included. MC was considered to affect a lumbar disc if at least one of its endplates were affected.

#### Grading of disc degeneration

Disc degeneration has been evaluated earlier using Pfirrmann classification^23^. The classification consists of progressive evaluation of disc degeneration, assessed from grade 1 (homogeneous disc with bright hyperintense white signal intensity and normal disc height) to grade 5 (inhomogeneous disc with hypotense black signal intensity and no more detectable difference between the nucleus and annulus). In accordance with previous studies^19,20^, grade 4 or greater was considered an indicator of a degenerate disc.

### Statistical analysis

Inter-rater agreement for the coding of endplate defects was calculated using Pearson’s correlation and Cohen’s weighted kappa as reported previously^20^. Critical scores were calculated for MC on the same lumbar level using Receiver Operating Curves (ROC)-analysis. Cumulative link mixed models and linear mixed models were used to analyse association between endplate defects and MC, adjusting for covariates and family structure. Kaplan-Meier survival analysis and Cox proportional hazards models analysis were carried out to infer any age-dependent relationship between endplate defect and MC. All calculations were performed in R using packages “psych”, “survival”, “ordinal”, “lme4”, and “OptimalCutpoints”.

## Results

Table 2 summarises the twin sample and its characteristics. In total, there were 831 subjects (mean age 54 ± 8 years, 95.8% female) in the study. Male subjects in the sample were similar to females as regards age and prevalence of MC, but they had significantly higher TEP scores in the lumbar spine compared to females (median [IQR] 29 [20, 36] vs 22 [19, 28], p = 0.001 respectively).

**Table 2:** Characteristics of the sample from TwinsUK.

Zygosity	N	Age (mean ± SD)	Age range	Sex (F/M)	BMI (median [IQR])	Pfirrmann sum score (mean ± SD)	TEP sum score (median [IQR])
MZ	242	57 ± 8	32–73	238/4	24.0 (21.9, 26.5)	16.2 (2.6)	23 (20, 29)
DZ	448	53 ± 8	19–71	420/28	24.2 (22.0, 27.1)	15.8 (2.6)	22 (19, 29)
Singleton	141	52 ± 9	35–73	138/3	23.8 (22.1, 26.4)	15.2 (2.7)	22 (18, 27)
Total	831	54 ± 8	19–73	796/35	24.1 (22.0, 26.9)	15.8 (2.7)	22 (19, 29)

BMI = body mass index, DZ = dizygotic, identical twin, F = female, IQR = interquartile range, MZ = monozygotic, fraternal twin, SD = standard deviation, TEP = total endplate.

### Endplate defects and Modic change

The prevalence and distribution of endplate defect are presented in Table 3. In summary, grade 2 was the most prevalent grade and grade 6 was significantly more prevalent at the lower lumbar levels than upper (p < 0.001).

**Table 3:** The prevalence and distribution of vertebral endplate defects within the lumbar spine.

	L1 rostral	L1 caudal	L2 rostral	L2 caudal	L3 rostral	L3 caudal	L4 rostral	L4 caudal	L5 rostral	L5 caudal	Total
Grade 1	329 (39.6)	58 (7.0)	254 (30.6)	50 (6.0)	204 (24.5)	34 (4.1)	137 (16.5)	36 (4.3)	249 (30.0)	152 (18.3)	1503 (18.1)
Grade 2	260 (31.3)	600 (72.3)	353 (42.5)	605 (72.9)	427 (51.4)	630 (75.8)	419 (50.5)	606 (73.0)	278 (33.5)	433 (52.2)	4611 (55.5)
Grade 3	57 (6.9)	60 (7.2)	95 (11.4)	74 (8.9)	95 (11.4)	84 (10.1)	92 (11.1)	56 (6.7)	78 (9.4)	65 (7.8)	756 (9.1)
Grade 4	106 (12.8)	79 (9.5)	83 (10.0)	57 (6.9)	53 (6.4)	38 (4.6)	58 (7.0)	21 (2.5)	52 (6.3)	25 (3.0)	572 (6.9)
Grade 5	61 (7.3)	20 (2.4)	27 (3.3)	24 (2.9)	23 (2.8)	17 (2.0)	16 (1.9)	15 (1.8)	20 (2.4)	17 (2.0)	240 (2.9)
Grade 6	17 (2.0)	13 (1.6)	18 (2.2)	20 (2.4)	29 (3.5)	28 (3.4)	108 (13.0)	96 (11.6)	153 (18.4)	138 (16.6)	620 (7.5)

Values are presented as total counts (%) by endplates adjacent to disc levels.

**Figure 2:**
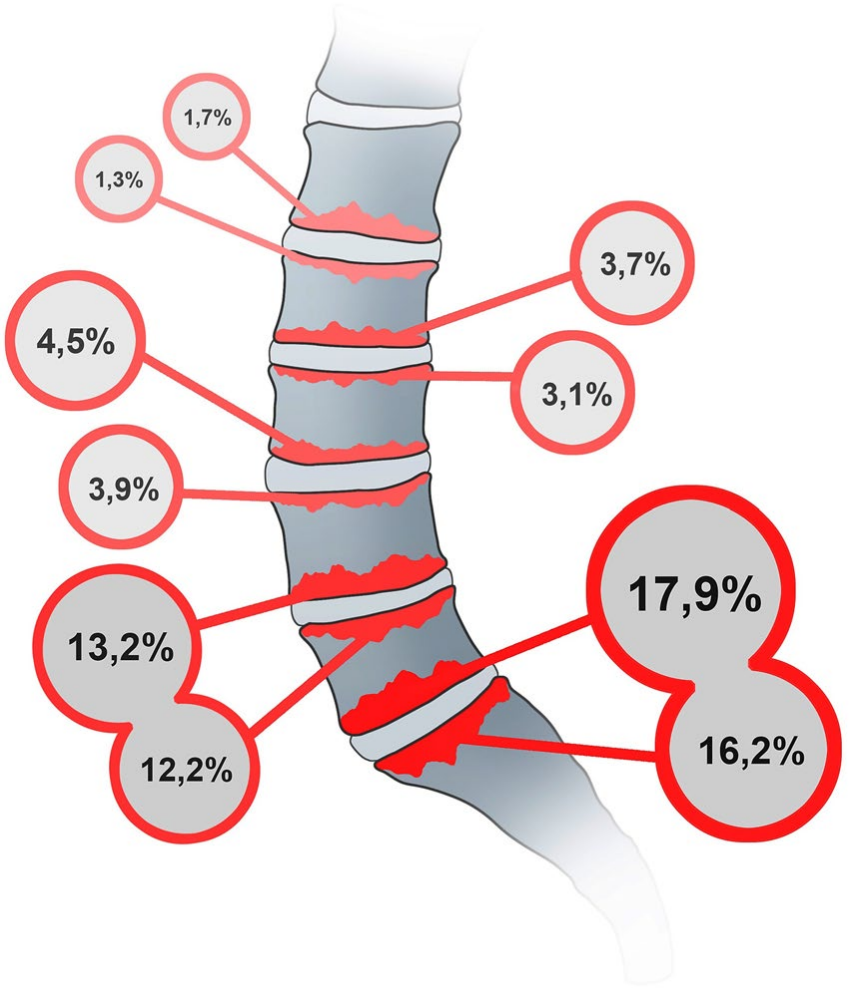
Prevalence and distribution of Modic change (MC) in the lumbar spine. The majority of MC is found in the lower lumbar spine (L4-S1) indicating a possible role of mechanical forces acting on the adjacent intervertebral discs and endplates of this anatomical region.

MC was present in 267 (32.1%) subjects. Of all the endplates being evaluated, we found 646 (7.8%) MC in total. Figure 2 shows the prevalence of MC by individual endplate levels. The prevalence of MC was significantly higher at the lower (L4-S1) compared to upper (L1-L4) lumbar levels (p < 0.001).

### Relationship between TEP score and MC

The prevalence of MC showed a significant correlation with increasing TEP score. Using multivariate linear mixed models to allow for covariates, TEP score was found to be strongly and independently associated with MC (risk estimates from 1.49 to 2.44 and p-values p < 0.001, at all lumbar levels, Table 4). Associations with other factors in the final multivariate models (including age, sex and BMI) were significant only at a few lumbar levels. ROC analysis indicated a critical TEP score of 6, above which there was a significantly higher likelihood of MC. Considering each lumbar level, similar cut-off points were found throughout the lumbar spine; TEP score of seven for L2/L3, and six for each of L1/L2, L3/L4, L4/L5, and L5/S1. Of all MC, 287 (81.3%) were at lumbar levels with TEP score ≥6 (Table 5).

**Table 4:** Risk factors for Modic change including endplate defects, by lumbar level.

Disc level	L1/L2, Estimate (SE)	p-value	L2/L3, Estimate (SE)	p-value	L3/L4, Estimate (SE)	p-value	L4/L5, Estimate (SE)	p-value	L5/S1, Estimate (SE)	p-value
TEP score	1.49 (0.31)	p < 0.001	1.54 (0.24)	p < 0.001	2.44 (0.31)	p < 0.001	2.03 (0.19)	p < 0.001	1.70 (0.21)	p < 0.001
Sex	−26.47 (854328.5)	0.999	−1.10 (1.24)	0.374	−1.18 (1.13)	0.293	−0.82 (0.65)	0.203	−0.40 (0.65)	0.542
Age	−0.02 (0.004)	0.569	0.002 (0.03)	0.947	0.01 (0.02)	0.635	−0.003 (0.02)	0.873	0.01 (0.02)	0.458
BMI	0.04 (0.06)	0.438	0.07 (0.04)	0.048	0.04 (0.04)	0.405	0.01 (0.03)	0.685	0.05 (0.03)	0.037

Association was assessed using multivariable models with adjustment for twin pairing. Sample size was 821. BMI = body mass index, SE = standard error, TEP score = total endplate score.

**Table 5:** Lumbar levels affected with MC distributed by total endplate (TEP) score <6 and ≥6.

	L1/L2	L2/L3	L3/L4	L4/L5	L5/S1	Total
TEP score <6	2 (12.5)	9 (18.1)	5 (12.5)	14 (12.3)	36 (23.8)	66 (18.7)
TEP score ≥6	14 (87.5)	23 (71.9)	35 (87.5)	100 (87.7)	115 (76.2)	287 (81.3)
Total	16 (100)	32 (100)	40 (100)	114 (100)	151 (100)	353 (100)

All values are presented as counts (%).

Regarding more precise endplate risk evaluation, the probability of having MC adjacent to the endplate having TEP score ≥6 was 6.4% at L1/L2, 12.2% at L2/L3, 21.9% at L3/L4, 48.5% at L4/L5, and 45.1% at L5/S1. Pearson’s correlation between TEP score and MC was statistically significant for every disc level. Considering the whole lumbar spine, if TEP score was ≥6 at any level, the probability of having MC at any level was 81.3%. At each lumbar level, there was a highly statistical significance in MC (p < 0.001) between those with and without TEP score ≥6 (except for L2/L3 TEP score ≥7).

**Figure 3:**
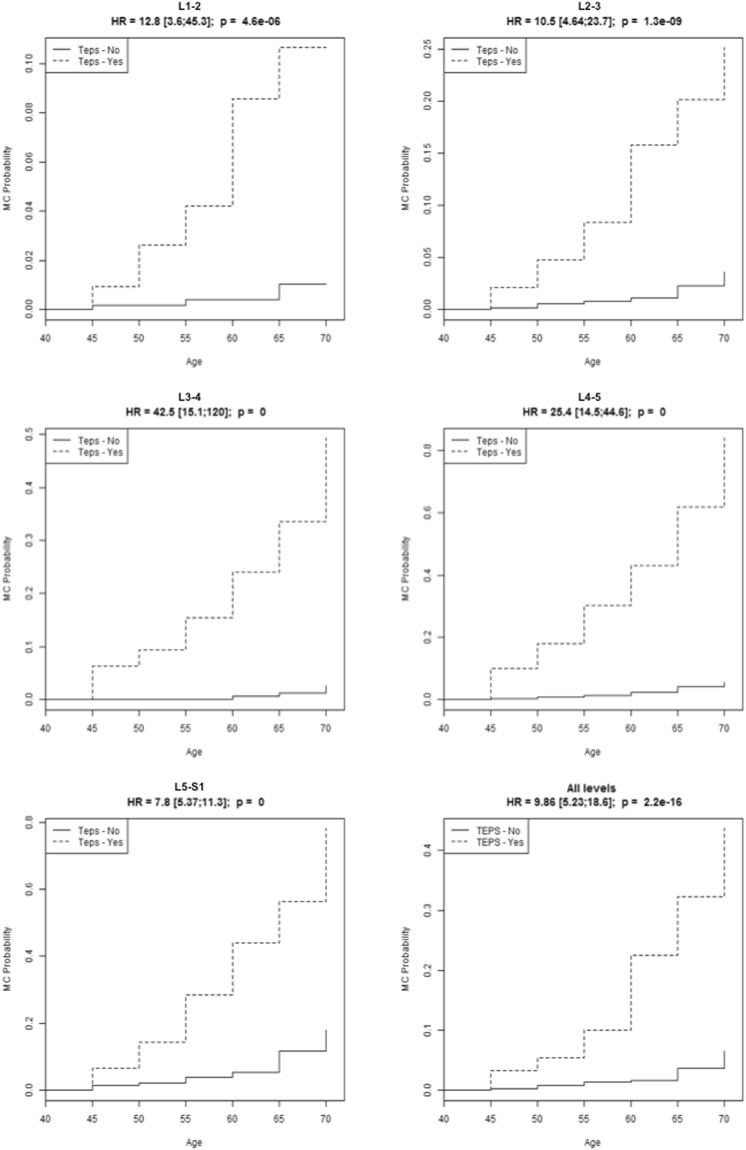
Survival analysis paired with Cox proportional hazards models analysis. Probability of having Modic change (MC) by TEP score ≥6 (denoted ‘Teps – Yes’ in the Figure) and <6 (denoted ‘Teps – No’ in the Figure). The probability is significantly increased in TEP score positive age subgroups at each disc level. The probabilities increase with age, and the influence of TEP score on MC is least at L5/S1. HR = hazard ratio.

A survival analysis paired with Cox proportional hazards model analysis provided the probabilities of having MC in the presence of TEP score <6 or ≥6, by age subgroups (Fig. 3). The probabilities increased significantly in TEP score ≥6 for all age strata.

## Discussion

This is the first large, population-based study examining the association between vertebral endplate defect and MC in the lumbar spine. Results showed that the presence of vertebral endplate defect in the lumbar spine was strongly associated with MC in the bone marrow adjacent to the endplate. When analysed by ROC, we found that a TEP ≥6 strongly predicted risk of MC.

Interestingly, our previous findings from the same sample from TwinsUK registry showed TEP ≥ 5 to be predictive for disc degeneration^20^ which may indicate that endplate defect, disc degeneration and MC are strongly associated with each other and it is highly likely that the same pathological process is involved. Moreover, our results are also in accord with the findings from another experiment in which endplate defect was correlated with DD in a clinical sample of 47 patients and 26 volunteers^19^. A cut-off score of 6 was found to be predictive of DD, after which a rapid response with a great increase of likelihood of DD for each unit increase in TEP score was shown, exactly as in our study with MC.

**Figure 4:**
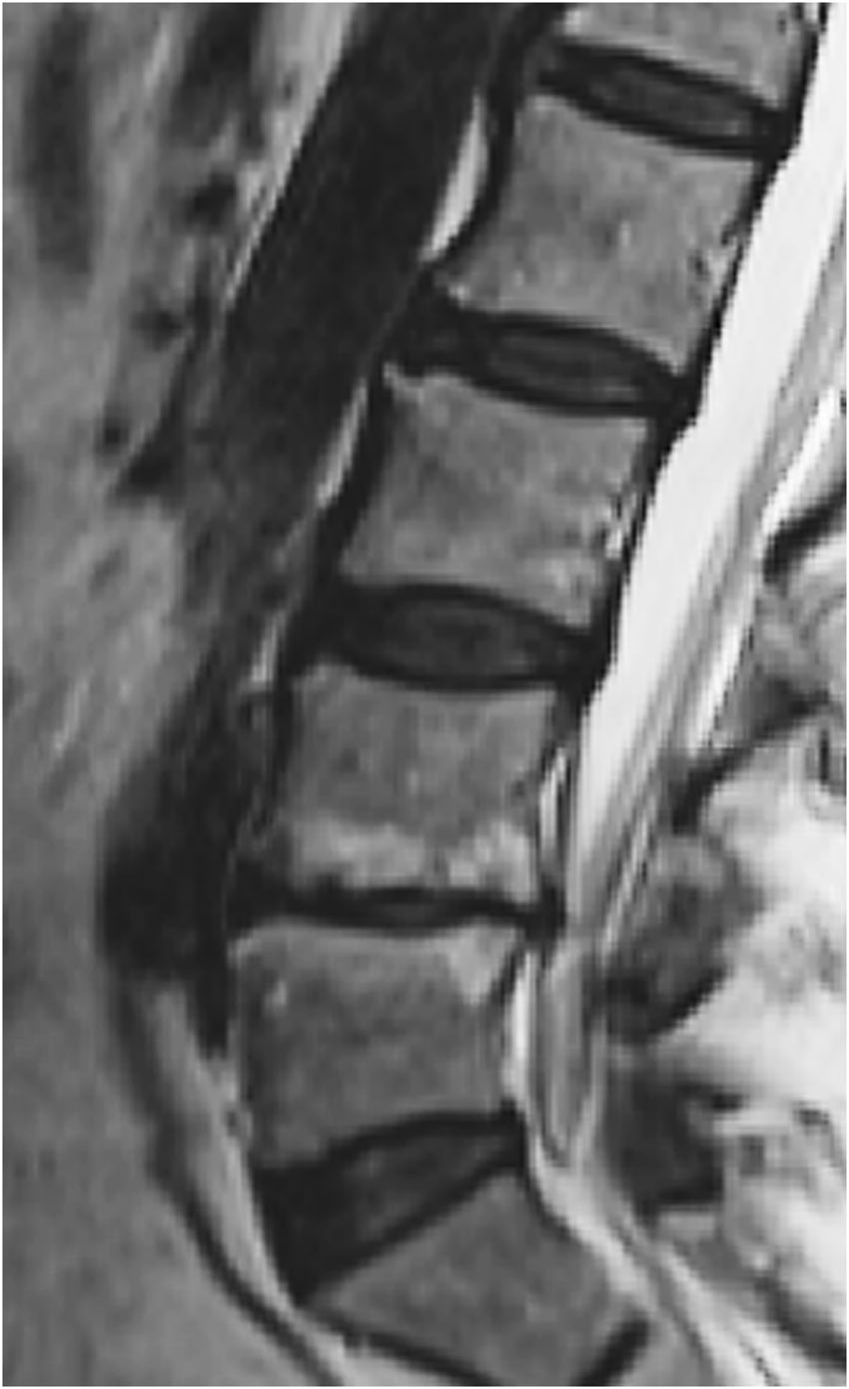
MRI scan showing endplate defect grade VI both at the L4/L5 rostral and caudal endplates, with associated Modic changes (MC) over both rostral and caudal bone marrows adjacent to endplates and disc degeneration evaluated as Pfirrmann grade 5. As the endplate is a fundamental part of the vertebral body-endplate-intervertebral disc motion segment, one could consider endplate defects to be an initiating factor not only for disc degeneration, but also for MC. MRI indicates magnetic resonance imaging. From Rade M, *et al*. Vertebral endplate defect as initiating factor in intervertebral disc degeneration: Strong association between endplate defect and disc degeneration in the general population. *Spine* **43**, 412–419 (2018). With permission.

In a clinical study with 1-year follow-up, MC type 1 was associated with both decreasing the disc height and increasing the size of endplate defects^17^. In another longitudinal clinical study sample DD, endplate defects and MC were found to be significantly associated with each other. Endplate defect grade ≥4 was found to be a risk factor for both DD and MC progression, with MC being the last MR feature to develop in this process^18^. All these results seem to build on the same line of thoughts (Fig. 4).

With regards to the motion segment in the spine, the cartilaginous endplate is considered to be a mechanically vulnerable structure^24^, which helps to equalize loading between the vertebral body and the disc^25^. At surgery, herniated disc material is reported to be found to contain cartilage endplate more frequently in those patients also manifesting MC^26^. Moreover, cartilage endplate stripping increases the permeability of the endplate and thus facilitates passage between the vertebral body and the intervertebral disc^26^.

That endplate defect may lead to both MC and DD can be explained by the physical location of the endplate; being positioned between the disc and vertebral body. Biomechanical studies suggest that microfractures at the vertebral endplate^27^ allow inflammatory cytokines to pass from the intervertebral disc to the bone marrow to initiate MC^28^, while any physical defect in endplate may allow transport of pro-inflammatory mediators from the disc to the vertebral body, leading to the oedema characteristically seen in MC^29^.

It is also plausible that an endplate defect creates the basis for low-grade virulent anaerobic bacteria (*Propionibacterium acnes, P. acnes*) to enter the disc from the vertebral body and give rise to slowly developing infection^28^. In this context, intervertebral disc infected with anaerobic bacteria have been shown to be more likely to develop adjacent vertebral body MC than those without^30^.

Biomechanically, endplate defect has been shown to lead to decompression of the adjacent intervertebral disc nucleus, which in turn could drive the degeneration of the disc through several mechanisms by (i) increase of shear forces acting on the annulus^31^; (ii) decrease of stability at that spinal level^32^ inducing disruptive changes in the annulus; (iii) altered matrix synthesis^33^; and (iv) vascularisation of the nucleus pulposus with autoimmune changes^30^.

There are some limitations we would like to highlight in this study. The TwinsUK sample shows a marked female predominance, for historical reasons. For that reason, every trait was examined separately by gender to determine how best to account for differences in the analysis. TwinsUK participants have been shown to be representative of singletons for a wide range of lifestyle and demographic traits^34^. For historical and funding reasons, only T2-weighted scans were available and, therefore, the evaluation of MC types was not possible. This study is cross-sectional by design, and our results support the hypothesis that endplate defect may be the initiating factor for MC and intervertebral disc degeneration. As a suggestion for further improvement we suggest designing a longitudinal study of endplates, DD and MC to unequivocably confirm the direction of such causality. Another relevant strength is the thorough training phase undergone by the two evaluators (JHM and MR) leading to almost perfect inter-rater agreement for endplate defect: kappa value of 0.86, Pearson correlation 0.864, with correlation and kappa being employed in conjunction to uncover nonrandom examiner error, as in Hunt^35^.

## Conclusions

To conclude, this study performed on a large population-based sample confirmed that endplate defect is strongly and independently associated with DD and MC, and that this relationship is evident in adults across the age spectrum and at all lumbar levels. As the same conclusions were reported for ED and DD in our previous study in the same population using the same methods, we imply that causal effects can occur between ED, DD and MC. A longitudinal study of twins’ MR spine scans and endplate is currently under way to better confirm the order of events.
